# Blasting extrusion pretreatment of sweet sorghum bagasse for enhanced enzymatic saccharification and ethanol production using *Pichia kudriavzevii* ATCC 20,381

**DOI:** 10.1186/s40643-025-00905-5

**Published:** 2025-06-19

**Authors:** Benjamín Vázquez-Rodríguez, Erick Heredia-Olea, Adriana Alamilla-Morales, Esther Pérez-Carrillo, David A. Perez-Perez, Sergio O. Serna-Saldívar

**Affiliations:** Escuela de Ingenieria y Ciencias, Tecnologico de Monterrey, Avenida Eugenio Garza Sada, Sur, Monterrey, 2501, CP 64849 N.L México

**Keywords:** Yeast, Lignocellulosic biomass, Screws configuration, Carbohydrate release, Biofuel

## Abstract

Blasting extrusion pretreatment (BEP) was evaluated for obtaining sugar enriched saccharified extracts from sweet sorghum bagasse (SSB) for ethanol fermentation. A factorial design was used for studying the effect of last barrel zone temperature (T_LBZ_) and screw configuration (SC) on carbohydrate release and extraction yield. The experiment with a higher total carbohydrate release was selected (BEP + ES) for a posterior 72 h fermentation and compared against enzymatic saccharified (ES) SSB without using BEP. Fermentation was performed at 28 °C, pH 5.0 and 10% of solids loading (BEP + ES and ES) using the stress tolerant yeast *Pichia kudriavzevii* ATCC 203,081 for ethanol production. In BEP experiments, screw configuration with one polygon element and a shear zone consisting of 7 reverse elements operating at a T_LBZ_ of 190 °C resulted in the highest carbohydrate release after enzymatic saccharification, achieving a 3-fold increase compared to control. BEP enhanced carbohydrate availability and lignocellulosic biomass degradation activity (enzymatic saccharification and *P. kudravzevii* fermentation). Ethanol production usining *P. kudriavzevii* with BEP + ES was 8.65-fold higher than the ES control after 72-h fermentation. Higher initial total carbohydrate (3-fold) and fermentable amino nitrogen (FAN) in BEP + ES (12.41-fold higher) improved SSB fermentability and thus ethanol yield. Glucose was fully consumed in the BEP + ES fermentation, while 91.62% was consumed in the ES experiment. Xylose and mannose/arabinose consumption varied by treatment, but *P. kudriavzevvi* displayed the ability to co-utilize pentoses and hexoses during fermentation. Compared to previous traditional twin-screw extrusion and ES, BEP enhanced SSB carbohydrate release during enzymatic saccharification, and carbohydrate consumption and ethanol production during fermentation.

## Graphical Abstract

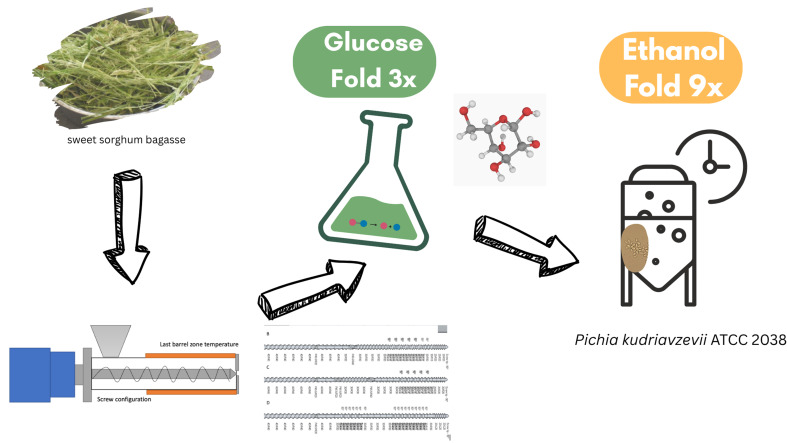

## Introduction

The global dependence on fossil fuels has instigated a critical reassessment of the energy landscape due to the associated environmental and economic consequences. As a result, carbon emissions reduction has become a paramount objective for nations worldwide. Bioeconomy, referred to as biobased economy, encompasses the production of biobased resources and their conversion into food, feed, bioenergy and biobased materials. A biobased value chain includes the primary production of biobased resources, their conversion to higher-value goods via processing and commercialization on the market (Zörb et al. 2018). Biorefinery acts as a strategic mechanism for the realization of circular bioeconomy. Sweet sorghum (*Sorghum bicolor* (L.) Moench) has emerged as a promising energy crop in this transition. Characterized by a rapid growth rate (C4 plant), high biomass yield, and significant CO_2_ sequestration capacity (Huang et al. 2023), sweet sorghum addresses key aspects of sustainable energy production.

This highly versatile energy crop provides a solution to the enduring food versus fuel debate, presenting stalks with a sugar-rich juice similar to sugarcane, composed of a di- and monosaccharide mixture of sucrose, glucose, and fructose (Umakanth et al. 2018). Beyond its role in yielding grain, sweet sorghum offers a comprehensive resolution: the grain serves dual purposes as both food and animal feed, while the sugars extracted from the stalks find valuable applications in direct fermentation (Bakari et al. 2023). The resulting byproduct, bagasse, assumes multifaceted roles, serving as fodder for heat generation, a lignocellulosic feedstock for second-generation biofuels (Heredia-Olea et al. 2015), and a source of biogas in anaerobic digesters (Machineni and Anupoju 2023; Prask et al. 2023). This unique versatility effectively reconciles the perceived conflict between food and fuel, where using lignocellulosic biomass that it is not edible to generate energy will not impact food security; aligning seamlessly with the essential requirements for food, fuel, and fodder in a sustainable and scientifically grounded framework. Sweet sorghum bagasse has a highly recalcitrant lignocellulose structure that hinders enzymatic and microbial degradation, limiting biofuel production (Velmurugan et al. 2019). Several factors impact the SSB composition, it can fluctuate between different sorghum varieties, but also the soil composition and the ecological conditions can affect the cellulose, hemicelullose and lignin composition in SSB plant cell walls, so an effective pretreatment (physical, chemical, biological, or hybrid) is crucial to disrupt this barrier. Advanced and combinational methods such as blasting extrusion pretreatment, are key for improving lignocelullosic biomass saccharification efficiency.

The extrusion process emerges as a cornerstone in the pretreatment of lignocellulosic biomass, serving as a crucial step in making the recalcitrant structure of sweet sorghum bagasse (SSB) more susceptible to subsequent processing (enzymatic hydrolysis or fermentation). This pretreatment method has a higher economic potential due to its low environmental impact and ability to operate at very high solids content, reducing water consumption once its mechanical and thermal effect on lignocellulosic biomass is characterized (Duque et al. 2017). The commercial availability and scalability of extrusion technology gives it a serious advantage over other well-established and novel emerging pretreatment methods, solidifying its consolidation in the field. Extrusion technology capability of processing biomass under mild temperatures, with practically no wastewater generated and negligible sugar loss contributes to its robustness and attractiveness (Li et al. 2019). This technology offers a continuous process with control over the type, frequency, and intensity of applied forces on the biomass, and can be used alone or combined with chemical or biological pretreatments (Karimipour-Fard et al. 2023). Extrusion has been demonstrated to be as effective as traditional alkaline and acid pretreatments, in terms of hydrolysis efficiency the saccharification yield of raw extruded soybean hulls compared with the material extruded and pretreated with acid and alkali lead to a 132% improvement in the saccharification yield over the raw material (Yoo et al. 2011). This study places particular emphasis on Blasting Extrusion Processing (BEP), BEP emerges as a recent branch of extrusion technology for food processing, a promising method with potential applications in the lignocellulosic biomass pretreatment industry. BEP involves high-pressure extrusion at low or standard temperatures, leading to rapid decompression and the transformation of insoluble lignocellulosic biomass (cellulose and hemicellulose) into soluble saccharides. (Li et al. 2012; Yan et al. 2015). This technique offers advantages over traditional methods, including high thermal efficiency, retention of heat-sensitive nutritional components, cost-effectiveness, and scalability. By combining physical phenomena like high pressure, shear stress, torque, and temperature (Gao et al. 2015). BEP has enhanced the conversion of dietary fiber (DF) to soluble dietary fiber (SDF) in food processing with remarkable results in carrot, soybean, and wheat residues, increasing up to 50% food SDF content (Chen et al. 2014; Gao et al. 2015; Orozco-Angelino et al. 2023; Yan et al. 2015). Additionally, CO_2_ blasting extrusion, achieved through the online generation of CO_2_ during extrusion, presents a promising alternative. This method, involving the blending of sodium bicarbonate and citric acid with materials, significantly increases pressure, leading to an over eightfold increase in soluble dietary fiber fraction in okara (Li et al. 2012). These controlled adjustments in energy distribution during BEP may promote a more uniform breakdown of lignocellulosic biomass, thus minimizing potential inconsistencies in the pretreatment process and allowing consistent results in subsequent enzymatic saccharification, as variations in the extrusion process can significantly impact the accessibility of enzymes to the structural components of SSB.

*Pichia kudriavzevii*, formerly known as (*Issatchenkia orientalis*, *Candida krusei* and *Saccharomyces krusei*) is a resilient yeast for diverse fermentation processes (food, biofuel and biotechnology applications) and has been applied in several traditional food fermentation processes such as natural wine fermentation (Chu et al. 2023; Zhu et al. 2020) and traditional sourdough production (Johansson et al. 2021; Kahve 2023). This non-conventional yeast exhibits unique characteristics such as a high thermotolerance (producing up to 200 g/L of ethanol at 45 °C) and resistance to diverse lignocellulosic fermentation inhibitors like acetic acid (70–100 mM), 5-hydroxymethyl furfural (5-HMF, 2–7.5 g/L) and furfural (15–30 mM) (Ndubuisi et al. 2020, 2023; Ruyters et al. 2015; Sugiyama et al. 2018). This thermotolerance, its tolerance to fermentation inhibitors and its high ethanol production capacity in such conditions, make *P. kudriavzevii* a candidate to intensify the bioethanol production by unifying saccharification and fermentation steps.

The objectives of this research were to study the relation between specific mechanical energies and its composition changes in SSB during BEP, by changing the extruder screw configuration in conjunction with the last barrel zone temperature, and its subsequent impact in enzymatic saccharification and fermentation efficiency for ethanol production. This study aimed to provide valuable insights into the process conditions for an extrusion-based pretreatment, contributing to the development of more targeted and efficient bioethanol production processes. The use of a BEP offers customizable means to enhance the overall bioconversion efficiency of SSB, adding versatility and adaptability to the thermoplastic extrusion process.

## Materials and methods

### Materials

Sweet sorghum (*Sorghum bicolor* (L.) Moench) was obtained from Instituto Nacional De Investigaciones Forestales, Agricolas y Pecuarias (INIFAP) C. E. (Rio Bravo, Tamaulipas, coordinates 25°57’55’’N, 98°01’02’’W). The sweet sorghum was transported to the Tecnológico de Monterrey (Nuevo Leon, Mexico) and the stalks were crushed three times through a fluted roller mill to extract the sweet juice and obtain the spent bagasse. Crushed stalks, leaves and panicles were dried at 60 °C for 24 h before being milled in a hammer mill (Willy Mill equipped with a 2 mm sieve).

### Physico-chemical characterization

The physical-chemical characterization of the SSB was conducted as was reported in previous work (Heredia-Olea et al. 2012). Moisture content was calculated according to the AACC 44 − 15 standard assay. Cellulose, hemicellulose, lignin, and structural sugars were quantified according to the NERL procedures (Sluiter et al. 2008).

### Blasting extrusion pretreatment

A twin-screw co-rotating extruder (BCTM-30, Bühler AG, Uzwil, Switzerland) with a barrel composed of 7 zones, a gravimetric feeder for solids, and a pneumatic feeder for water was used. The temperature of the two last barrel zones was controlled using two heat exchanger devices (Tool Temp, Bühler AG, Uzwil, Switzerland). One heat exchanger controlled the zones 4–5 and the other device the zones 6–7. Two modular screws were used with a length of 1.040 mm, 30 mm diameter and a L/D ratio = 28. A die with a single circular 4 mm hole was used. A fly nozzle was set at the end of the extruder barrel, setting the die 2 mm before touching the screw tips. A thermocouple was set at the last chamber of the barrel to control this section temperature; another thermocouple and pressure sensor were set inside the fly nozzle to measure the output conditions of the extrudates.

### Screws configurations

**Fig. 1:**
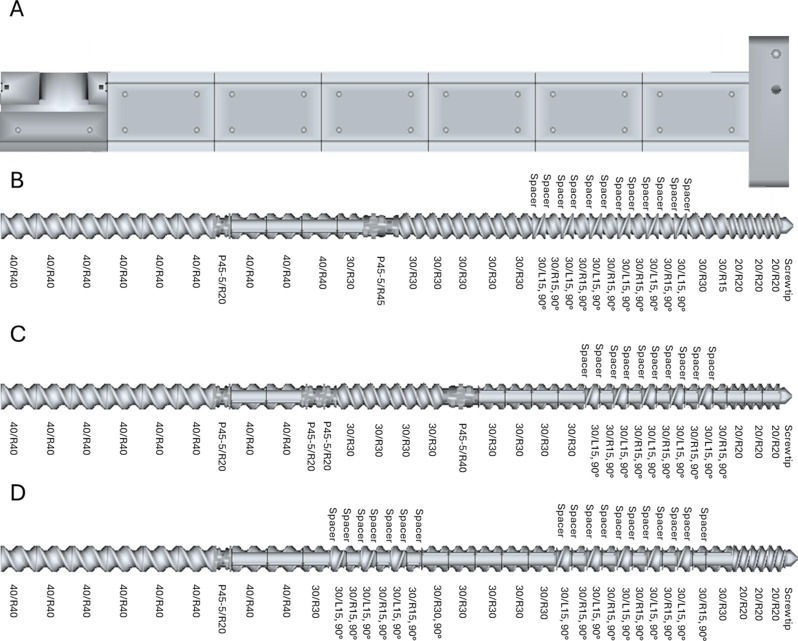
Different screw configurations and barrels used to extrude the sweet sorghum bagasse. A- extruder’s barrel with seven zones, the heat exchangers were set at the two last zones neat to the endplate; B- screw configuration with 1 polygon element and 1 shear zone composed by 7 reversal elements (configuration 1); C-screw configuration with 1 polygon element and 1 shear zone composed by 5 reversal elements (configuration 2); D- screw configuration with 2 shear zones, the first at the middle of the screw composed by 3 reversal elements and the second with 5 reversal elements (configuration 3)

Three different screw configurations (Fig. 1) were tested to enhance high shear stress. Configurations varying the number of reverse elements, polygon elements and their position on the screw’s shaft. The solids feed rate was adjusted to avoid blocking the extruder due to excess torque. The water percentage was adjusted to 36% for all tests using the extruder feeding system with a pnematic pump (IP67/NEMA/Type4x, Endress + Hauser, Switzerland).

### Enzymatic saccharification

The pretreated sweet sorghum bagasse was hydrolyzed (SSB) using a total reaction volume of 100 mL prepared in 500 mL Erlenmeyer flasks using a solid loading of 10% w/v. Pretreated sweet sorghum bagasse was hydrolyzed using the enzymes Cellic^®^ CTec2 and Cellic^®^ HTec2, dosed at 5.4% and 0.6% of the total amount of cellulose. The aliquots were centrifuged at 4500 rpm and filtered through a 0.22 𝜇m filter. The total sugars, inhibitors, and ethanol of the filtered samples were analyzed by HPLC while the free amino nitrogen (FAN) was determined after reaction with ninhydrin. The amount of FAN was determined in a spectrophotometer set at 420 nm. The calibration curve was constructed using glycine as standard (Heredia-Olea et al. 2015).

### Fermentation

Two fermentation experiments were performed, first one was sweet sorghum bagasse saccharified using enzymatic saccharification (ES) without extrusion treatment (control) and extruded sweet sorghum bagasse with BEP E_1_ experimental conditions coupled with saccharification enzymes (BEP + ES). Fermentation was performed using the yeast *Pichia kudriavzevii* (strain *Issatchenkia orientalis Kudrjanzev* ATCC 20381). Experiments were performed by triplicate in 500 mL Erlenmeyer flasks with 225 mL of the hydrolysates. Beforehand, the strain was incubated in Difco YM broth (Becton, Dickinson and Company, USA) in an orbital shaking incubator (VWR Model 1575) set at 28 °C and 100 rpm. The hydrolysates were adjusted to 10% solids with citrate buffer pH 5. A concentration of 1 × 10^6^ cells⋅mL^− 1^∘Brix^− 1^ was inoculated into each prepared reaction flask. Aliquots of the fermentation broth were taken at 0, 24, 48, and 72 h of fermentation. The aliquots were centrifuged at 4500 rpm and filtered through a 0.22 𝜇m filter. The total sugars, inhibitors, and ethanol of the filtered samples were analyzed by HPLC while the free amino nitrogen (FAN) was determined after reaction with ninhydrin. The amount of FAN was determined in a spectrophotometer set at 420 nm. The calibration curve was constructed using glycine as standard (Heredia-Olea et al. 2015). The fermentation was performed without any nutrient supplementation at 28∘ C in an incubator (VWR Model 1575) under anaerobic conditions.

### HPLC quantification

The fermentation and enzymatic saccharified samples were treated as described in previous research (Heredia-Olea et al. 2015). Analytes were separated by a Shodex SH1011 column (300 × 7.8 mm) with a flow rate of 0.6 mL per minute of HPLC-grade water containing 5 mM H_2_SO_4_ for the quantification of inhibitors and ethanol. The sugar quantification was performed with a Shodex SP0810 column and a cation/anion deasher (Biorad). The column temperature was set at 60 and 85∘ C for inhibitors and sugars, respectively. Also, the detector was at 50∘ C and the autosampler (refractive index detector Waters 2414) was at 4 °C. Standards of ethanol, cellobiose, D-glucose, D-xylose, L-arabinose, D-mannose, D-galactose, acetic acid, 5-hydroxymethylfurfural, and furfural (Sigma Chemical Co., St. Louis, MO) were used. The run times for sugars and inhibitors quantifications were 20 and 45 min, respectively.

### Experimental design and statistical analysis

All blasting extrusion experimental treatments (E_1_, E_2,_ etc.) are shown in Table 1. The processing parameters evaluated prior enzymatic saccharification efficiency were screw configuration (SC), last barrel zone temperature (T_LBZ_), product temperature (T_P_), specific mechanical energy (SME), relative torque (R_T_) and end plate pressure (P) all data generated by the Extruder software VWS_BCTBII (Bühler AG, Uzwil, Switzerland). A factorial design using three different SC levels and a high and low level for T_LBZ_ was evaluated as shown in Table 2. Data was subjected to a One-way analysis of variance with a Tukey’s multiple comparison test, and differences among means were compared with Tukey’s tests with a level of significance of *p* < 0.05.

**Table 1:** Extrusion parameters obtained of the six different pretreatments extruding sweet sorghum Bagasse (SSB)

Parameter	Pretreatments					
	SC_1_		SC_2_		SC_3_	
	E_1_	E_2_	E_3_	E_4_	E_5_	E_6_
Last barrel zone temperature (°C)	190	130	186	135	185	130
Product Temperature (°C)	118	87	94	116	97	109
Endplate Pressure (bar)	28	112	77	72	88	91
SME (Wh/kg)	439	944	518	878	544	961
Screws speed (rpm)	201	201	201	201	201	201
Relative Torque (%)	43	90	47	76	45	91
Solid feeding rate (kg/h)	3.81	3.84	3.83	3.49	3.50	3.96

**Table 2:** Independent variables and their levels used for the SSB BEP factorial design

Independent Variable	Factorial level		
Screw Configuration (SC)	1	2	3
Last barrel zone temperature (T_LBZ_)	High Temperature	Low temperature	-

All fermentation experiments shown in Table 3. A factorial experiment was design considering the type of treatment (BEP + ES and ES) and the fermentation time (0, 24, 48 and 72 h) as independent variables, the different carbohydrate concentration extracted from during the fermentation experiments was subjected to a One-way analysis of variance with a Tukey’s multiple comparison test, and differences among means were compared with Tukey’s tests with a level of significance of *p* < 0.05. The Pearson correlation test between the response variables in saccharification and fermentation experiments was calculated with the pairwise method using statistical software Minitab.

**Table 3:** Independent variables and their levels used for the ethanolic fermentation factorial design

Independent Variable	Factorial level			
Type of pretreatment	BEP + ES	ES	-	-
Fermentation time (h)	0	24	48	72

## Results and discussion

### Physico-chemical characterization

Results of the characterization of the SSB are shown in Table 4. The SSB contained 32.05% ± 0.28 of glucans, 17.45% ±1.00 of xylans and 1.99% ± 0.02 of arabinans, 0.51% ± 0.04 of soluble lignin, 16.76% ± 0.19 of insoluble lignin. This sugar composition indicates a higher cellulose composition than hemicellulose present in SSB. This lignocellulosic material sugar composition was similar to previous sweet sorghum bagasse research with values ranging from 36.5 to 37.1% for glucan, 17.7 to 18.2 for xylan and 7.4 to 29.10 for lignin (Heredia-Olea et al. 2015; Punia and Singh 2024; Su et al. 2020; Wen et al. 2018). Protein and ash content were also determined as shown in Table 4, being 3.40 ± 0.17% and 6.73 ± 0.45% respectively; these percentages might be considered low for viable ethanol production, as lignocellulosic biomass with > 40% cellulose content are regarded as potential feedstocks for bioethanol production (Malik et al. 2022).The environmental influence in the expression of lignocellulosic biomass composition can be related to the wide range of glucan, xylan, and lignin composition presented in different studies; factors such as the difference in edaphoclimatic variations between the cultivar environments during the growing season; accumulated precipitation; temperature; severity of diseases that affect the culture, may impact the final composition of the cellulose and lignin present in SSB up to 5 and 3%, respectively and therefore affecting its viability as a raw material for second generation ethanol production (Almeida et al. 2019).

**Table 4:** Physical-chemical characterization of sweet sorghum Bagasse (SSB)

Component	Percentage (dry basis)
Water soluble extract	13.37 ± 0.30
Ethanol soluble extract	1.74 ± 0.01
Glucans	32.05 ± 0.28
Mannans/Arabinnans	1.99 ± 0.02
Xylans	17.45 ± 1.00
Acetate	3.18 ± 0.03
Insoluble lignin	16.76 ± 0.19
Soluble lignin	0.51 ± 0.04
Ashes	6.73 ± 0.45
Protein	3.40 ± 0.17

### Blasting extrusion pretreatment and enzymatic saccharification

**Fig. 2:**
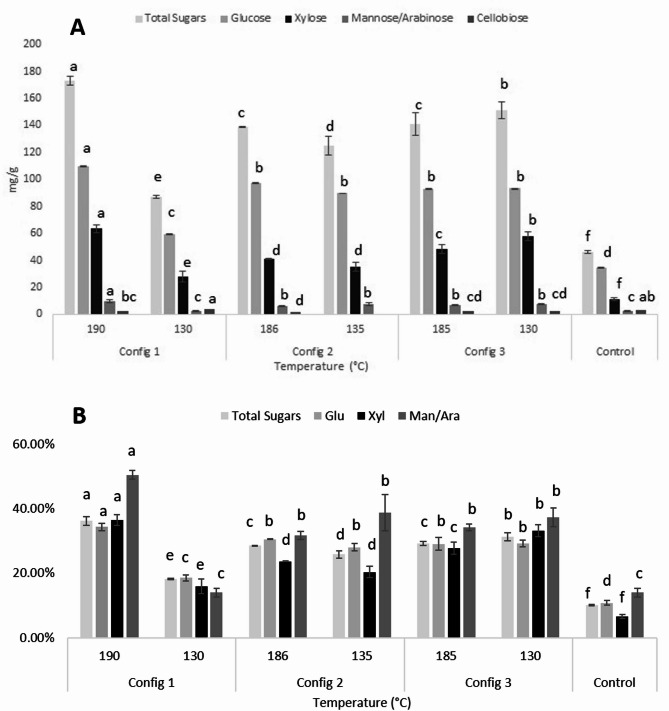
Sugar extraction (**a**) and enzymatic saccharification yield (**b**) from SSB bagasse and SSB extrudates blasting extrusion processing. The carbohydrate extraction is expressed as milligrams of sugar per gram of SSB dry basis. Means with different letters within columns denote significant median differences by Tukey-Krammer test (*p* ≤ 0.05). Hydrolysis yield was calculated as: (grams of monosaccharide obtained after enzymatic hydrolysis/ grams of total structural monomeric sugars in raw material)*100

The most common equipment for biomass pretreatment is the full-intermeshing co-rotating twin-screw extruder. In co-rotating twin-screw extruders, the shearing and plasticizing effect is axial (the maximum velocity is achieved at the intermeshing zone), while in counter-rotating equipment, the effect is radial (the highest velocity is achieved at the screw tips). The use of co-rotating twin-screw extruders has several advantages such as better heat and mass transfer capabilities, good control of residence time distribution and mixing. Moreover, full-intermeshing co-rotating twin-screw extruders could operate at smoother conditions, while producing high shearing forces along the process (Duque et al. 2017). Twin-screw extrusion enhances material processing by utilizing two intermeshing screws, optimizing temperature control and mixing efficiency, making it widely applicable in plastics, pharmaceuticals, and specialty food industries. In contrast, blasting extrusion integrates intense mechanical disruption, effectively breaking down the recalcitrant lignocellulose matrix in biomass. This approach significantly improves sugar recovery and fermentation efficiency, surpassing conventional twin-screw methods for biofuel production. Variations in sugar composition also reflect the impact of screw configurations. All extruder screw configurations (SC) increased sugar release during enzymatic hydrolysis (Fig. 2). The SC selected for the extrusion pretreatment (Fig. 1) process aligns with previous studies that have highlighted the importance of optimizing screw design for enhancing biomass conversion efficiency. Studies byGu et al., (2019) andKuster Moro et al., (2017) have shown that adjusting screw configurations can impact residence time and mixing efficiency within the extruder, affecting the overall effectiveness of pretreatment processes. By avoiding blockages due to excessive torque, as highlighted in the research by(Karunanithy and Muthukumarappan 2011) the right screw configuration ensures a continuous and smooth flow of biomass material during extrusion.

Treatments with SC_1_ (E_1_ and E_2_) exhibited significantly different endplate pressures most likely due to last barrel zone temperature (T_LBZ_) variations. E_1_, with a high T_LBZ_ (190 °C), exhibited a low endplate pressure of 28 bar. This decrease in endplate pressure can be attributed to the reduced viscosity of the lignocellulosic material at higher T_LBZ_, which facilitates material flow and results in lower pressure buildup. The screw design of the latter experiments included one polygon element and one shear zone (composed of seven reverse elements), which offer less resistance to material flow; thus, contributing to the low endplate pressure. These results align with the findings of Sandrin et al. (2018), who noted that higher T_LBZ_ reduces material viscosity and endplate pressure. In contrast, E_2_, operating at a lower T_LBZ_ of 130 °C, showed a much higher endplate pressure of 112 bar. The lower T_LBZ_ likely maintained a higher material viscosity, increasing resistance and pressure. This finding is consistent with other reports which highlighted that lower T_LBZ_ results in higher viscosities and endplate pressures during extrusion (Guiao et al. 2022).

Polygon or kneading block functions as the primary mixing element, thus generating complex velocity fields composed of strong components both in the cross-sectional plane of the mixer and backwards and forwards along the rotation axis. Polygon element was present in SC_1_ and SC_2_ and was removed from SC_3_ (Fig. 1). The velocity field around the kneading blocks generates strong mixing in both directions with the axial direction being the largest (Robinson and Cleary 2019), thus generating a plasticizing effect on the extruded material. The extension of the shear zone from five to seven reverse elements increased yield but the presence of a polygon block might be needed according to data obtained from SC_3_.

Another parameter of the extrusion-cooking process that was tested was its energy consumption intensity. E1 treatment had the lowest endplate pressure and specific mechanical energy (SME). The highest SME value was 961 Wh/kg and was obtained with E6; the lowest SME obtained was during E1 and was 439 Wh/kg. Energy consumption is one of the main concerns of biomass pretreatment, therefore the recovery of the highest achievable amount of product under the lowest possible energy consumption conditions (Rezania et al. 2020). The torque is inversely proportional to the screw speed, so the mechanical energy is lower at higher screw speeds. As with every continuous process, a certain time is required in extrusion before a continuous regime is established, torque values tend to peak quickly and fluctuate during the first moments of operation (Duque et al. 2017). This can be attributed to the intermittent formation of discrete fiber plugs in the zones with functional screw elements (kneading blocks and reverse flux elements) until the steady-state is achieved. There is a negative relationship between temperature (thermal energy) and torque; higher temperatures lead to lower torque requirements, due to the softening of biomass and decrease of viscosity (Duque et al. 2017). The moisture content of the feed (liquid-to-solid ratio inside the extruder) impacts greatly the torque, several previous studies reported increments in torque as biomass moisture content decreases (Duque et al. 2017). Although torque is an adequate parameter to measure the mechanical energy of an extruder, torque values vary greatly depending on the size of the equipment, the type of biomass extruded, and the operational conditions; to facilitate the comparison among the different pretreatments, a new parameter that considers the throughput of the process is used: the specific mechanical energy factor (SME). The SME measures the mechanical energy consumption in the extrusion treatment by estimating the mechanical energy required to produce a unit weight of pretreated biomass.

Total carbohydrate release ranged from 173.31 ± 3.32 to 46.02 ± 1.01 mg/g SSB (36.13% ± 1.34–10.10% ± 0.24% of hydrolysis yield) (Fig. 2a) and was significantly impacted by both T_LBZ_, SC and their respective interaction (Table 5). The highest carbohydrate yield was 36.13% and was obtained when extruding the SSB at 190 °C and using SC_1_. Except for E_6_, increments in T_LBZ_ favored total carbohydrate release from SSB lignocellulosic matrix to the extraction solvent, as shown in Figure. 2a. Control treatment released 10% of the total carbohydrates present in SSB. Using the BEP, total carbohydrate release was enhanced by 26.03%, an 11.46% increment from previous results using traditional extrusion pretreatment as pretreatment for SSB (Heredia-Olea et al. 2015). The differences in all experiments are a result of the variance in thermal and high shear energy produced among the experiments; the use of high shear energy in lignocellulosic biomass pretreatments, particularly through the use of reverse screw elements in twin-screw extruders, has been shown to significantly enhance the carbohydrate extraction yield from lignocellulosic biomass (Del Lopez et al. 2019). Higher temperatures during pretreatment generally enhance enzymatic digestibility by breaking down hemicelluloses and lignin, making cellulose more accessible for enzymatic hydrolysis and thus increasing total carbohydrate release. For instance, enzymatic digestibility of wheat straw and rapeseed straw significantly increased at elevated temperatures, with optimal results typically observed around 190–205 °C (Choi et al. 2013). This effect is attributed to a more efficient breakdown of the complex lignocellulosic biomass structure, enhancing enzymatic activity on cellulose components and thereby increasing overall process efficiency in biomass conversion. The increase in enzymatic efficiency is all so related to porosity created in the lignocellulosic biomass by water evaporation under mild and high T_LBZ_ (above 100 °C) in extrusion processing. The increase in porosity results in an increase in the specific surface of the extrudate, which is highly beneficial for enzymatic hydrolysis (Konan et al. 2022).

**Table 5:** Analysis of variance of the selected processing parameters for the evaluated responses in SSB BEP + ES process. Carbohydrate release are expressed in Mg g^− 1^ of SSB

Factor	Total Sugars		Glu		Xyl		MAF		Cellobiose	
	p-value	R^2^	p-value	R^2^	p-value	R^2^	p-value	R^2^	p-value	R^2^
Screw Configuration (SC)	0.029*	0.9527	0.099	0.8899	0.008**	0.8953	0.435	0.8911	< 0.001**	0.9374
Last barrel zone temperature (°C)	< 0.001**		< 0.001**		0.005**		0.008**		0.699	
Interaction	< 0.001**		< 0.001**		< 0.001**		< 0.001**		0.001**	

* Significant at *p* < 0.05; ** highly significant at *p* < 0.01

Glucose release ranged from 109.8 ± 3.8 to 34.59 ± 2.1 mg Glu/g SSB (Fig. 2a). and was significantly impacted by T_LBZ_ and T_LBZ_*SC interaction (Table 5). The highest glucose release was 109.8 ± 3.8 mg Glu/g SSB and was obtained when extruding the SSB at 190 °C and using SC_1_ (E_1_). Control treatment released 10.79% of the glucose present in SSB. Using the BEP pretreatment, glucose release was enhanced by 26.03%, a 10.27% increment from previous results using traditional extrusion pretreatment as pretreatment for SSB (Heredia-Olea et al. 2015). Similar enhancement in cellulose conversion was obtained using twin-screw extrusion technology for corn-stover pretreatment, with a 12–28% increase in glucose released by enzymatic hydrolysis which might be related to the particle size reduction by extrusion pretreatment (Wang et al. 2020). These results contrast with chemo-mechanical twin-extrusion of blue agave bagasse performed by Montiel et al. (2024), where they report an increment of almost 50% cellulose recovery and glucose and xylose hydrolysis of 80 and 39% respectively after a twin extrusion pretreatment using NaOH and phosphoric acid as chemical assistants; in this study BEP showed and increase of 23% and 29.8% on the glucose and xylose hydrolysis without any chemical assistance, leaving room to potencial improvements using acid or alkaline treatments in conjunction with BEP. As shown in Fig. 2a, Glucose release showed no significant difference when using SC_2_ and SC_3_ and as T_LBZ_ increased (*p* > 0.05), from 135 °C to 186 °C, in E_4_ and E_3_ respectively; and 135 to 185 °C, in E_6_ and E_5_. The SC greatly affects glucose release due to its impact on the lignin redistribution among the lignocellulosic matrix, an increase in mechanical severity was expected on SSB when using SC_2_ and SC_3_ as they varied the number of reverse elements as well as the number of shear zones along the screw, previous results on the twin-screw extrusion of corncob showed that a blockage of pores on the fiber can occur through lignin surface rearrangement when using a high number of reverse and kneading screw elements leading up to a 5% of glucose release reduction, also making lignin more susceptible to alkaline chemical pretreatments to further enhance glucose release (Zheng et al. 2015). Results in E_1_ also suggest that high T_LBZ_ conditions favor the hydrolysis of cellulose to glucose, this finding is supported by studies that demonstrate the beneficial effects of high temperatures on hydrolysis and sugar release (Álvaro H.M et al., 2022). On the other hand, results produced in E_2_ indicate that a lower T_LBZ_ was not as effective in converting cellulose to glucose despite greater friction and shear produced by the SC.

Cellobiose sugar was significantly impacted by SC and T_LBZ_*SC interaction (Table 5), treatments with SC_1_ didn’t have a significant difference when compared to the control experiment (Fig. 2). The presence of cellobiose in E_2_ is higher than in all the other experiments, in the case of E_4_ its presence wasn’t detected (Fig. 2). This lack of cellobiose and its low concentration on the E_2_ and E_4_ indicate that BEP had a beneficial impact on β-glucosidase productivity present in Cellic CTec2 by enhancing the cellulose conversion to glucose.

Xylose release ranged from 62.52 ± 2.96 to 11.43 ± 1.07 mg Xyl/g SSB (Fig. 2a) and was significantly impacted by both T_LBZ_, SC, and their respective interaction (Table 5). The highest xylose yield was 62.52 ± 2.96 mg Xyl/g SSB and was obtained when extruding the SSB at 190 °C and using SC_1_. Xylose yield did not increase in all experiments when T_LBZ_ was increased, when using SC_2_ xylose yield showed no significant difference when increasing the T_LBZ_ from 135 °C to 186 °C, E_4_ and E_3_ respectively (Figure. 2a). When extruding with SC_3_, increasing T_LBZ_ from 130 °C to 180 °C reduced 16.25% the xylose release from SSB lignocellulosic matrix. Previously it has been reported that the number of reverse elements in the screw configuration impacts greatly the recovery of xylose from a lignocellulosic matrix. The higher number of reverse elements are present in E_1_ and E_6_ (SC_1_ and SC_2_, respectively) and may have impacted the release of xylose via different mechanisms, in E_1_ the effect of T_LBZ_ might have been the primary force behind the xylose release, whereas the recovery in E_6_ can be related to the increase in SME and endplate pressure derived from the reverse element present in the two shear zones along the screw shaft. Reverse elements importance in screw design is supported by (Zheng et al. 2016) results, this research obtained up to 80% of xylose recovery from steam-exploded corncobs by augmenting the number of reverse elements in present in the screw shaft using traditional twin-screw extrusion, thus augmenting end plate pressure up to 1136 kPa. Control treatment released 6.55% of the xylose present in SSB, using the BEP xylose release was enhanced by 29.85%, a 38.60% increment from previous results with SSB using traditional extrusion pretreatment (Heredia-Olea et al. 2015). Preceding, twin-screw extrusion has shown promising results as a pretreatment in biorefineries studies for successfully recovering and fermenting glucose and xylose for ethanol an xylonic acid production (Madadi et al. 2023), also twin extrusion in conjunction with enzymatic peeling processing for the release of glucose and xylose from wheat bran, by reducing enzymatic peeling time and increasing selectivity in the extraction of arabinoxylans from the lignocellulosic matrix (Hell et al. 2015) These previous mentioned studies and their results in enhancement of saccharification of glucose and xylose expose blasting extrusion pretreatment as a sustainable feasible and novel technology with increased sugar release when compared to emerging technologies that depend on elevated enzymatic hidrolisis times (up to 72 h) and/or the use of strong acid and alkali pretreatments such as the extrusion + ultrasound pretreatment (77.5% of glucose and xylose hemicellulose conversion) and chemo-mechanical extrusion (10% increase in cellulose and hemicellulose recovery and 65.9% of Glu and Xyl conversion after 24 h hydrolysis) (Cha et al. 2016; Shukla et al. 2023; Zhang et al. 2020). Xylose is the second most abundant sugar in lignocellulosic biomass but unlike glucose, it has not been used extensively by the energetic industry. However, in recent years the bioconversion of xylose into value-added chemicals has received a lot of attention, *Yarrowia lipolýtica* being a good example of xylose used as a fermentation substrate to produce succinic acid (Narisetty et al. 2022).

Mannose + arabinose (MA) release ranged from 10.03 ± 0.26 to 2.77 ± 0.26 mg MA/g SSB (Fig. 2a) and was significantly impacted by both T_LBZ_, SC and their respective interaction (Table 5). The highest MA extraction was 10.03 ± 0.26 mg MA/g SSB and was obtained when extruding the SSB at 190 °C and using SC_1_. MA yield presented a similar release behavior as Xylose, and it didn’t increase in all experiments when T_LBZ_ was increased. When using SC_2_ and SC_3_ MA release showed no significant difference when increasing the T_LBZ_ (Table 3). Control treatment released 14% of the MA present in SSB, using the BEP pretreatment, MA release was enhanced by 36% (Figure. 2a), a 38.60% increment from previous results with SSB using traditional extrusion pretreatment (Heredia-Olea et al. 2015). Mannose and arabinose being components of hemicellulose followed a similar release behavior as xylose after enzymatic saccharification, the difference in magnitude is attributable to SSB hemicellulose amount (32.57 ± 0.62% – 27.99 ± 0.40%) (Li et al., 2014) and its monosaccharide composition which can range from (14 to 0%) for mannose (Ray et al. 2018; Velmurugan et al. 2019) and 35.74 ± 0.73% to 9.33 ± 0.36% for arabinose (Qiu et al. 2017). These increments in mannse and arabinose extraction after BEP contrast with those reported previously (Álvaro H.M et al., 2022) for the extrusion pretreatment of corncob, where a 26.6% decrease in glucose release was obtained, this is mainly due to the simultaneous hemicellulose decomposition and production xylose and arabinose (using Cellic HTec2 enzyme mix), these attributed to the inhibition of cellulases present in the Cellic Ctec2 mixture by the extracted C5 sugars. Mannose and Arabinose have several new clinical and biomedical applications, for instance, mannose encompasses several bioactivities such as anti-inflammatory properties, pathogenic bacterial inhibitions, metabolism regulation, and suggested involvement in alleviating diabetes and obesity. Additionally, mannose shows promise in antitumor effects, immune modulation, and the construction of drug carriers, indicating a broad spectrum of therapeutic potential (Chen et al. 2024). Arabinose is renowned for its ability to inhibit sucrosei absorption and regulate glycemic effects by inhibiting sucrose. Moreover, L-arabinose has demonstrated potential in preventing constipation, improving glucose tolerance, regulating intestinal microorganisms, acting as a colitis inhibitor, and alleviating metabolic syndrome (Xiang et al. 2024).

### Ethanolic fermentation of SSB with BEP + ES and ES using Pichia kudriavzeyii ATCC 20,381

**Fig. 3:**
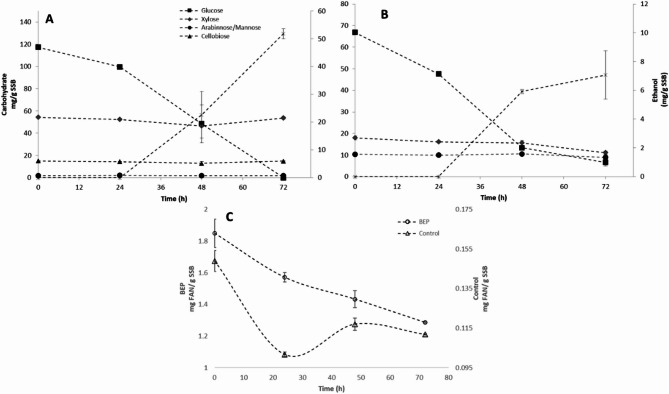
Comparison of the time-courses for *Pichia kudriavzevii* using sweet sorghum bagasse hydrolizate treatment E1 (**A**) and without treatment (**B**), and the FAN consumption in both experiments

Figure 3 shows the carbohydrate and FAN consumption obtained for the ethanolic fermentation of BEP + ES and enzyme saccharification (control) of SSB using *P. kudriavzevii*. The monosaccharides detected during the early stages of fermentation were related to the partial hydrolysis produced by the enzymatic saccharification of SSB structural polysaccharides (Figure. 3-B) and the interaction of the high energy of BEP and enzymatic saccharification of cellulose, hemicellulose and lignin (Figure. 3-A). The SSB structural polysaccharides were mainly composed of glucose, xylose, mannose and arabinose. After 72 h fermentation maximum ethanol concentration was obtained, BEP extrude and control fermentation produced 51.8435 ± 1.8265 and 5.99 ± 1.40 mg EtOH/ g SSB, respectively (Fig. 3-A and -B).

In addition to the effect on the plant cell wall structural polysaccharides, the BEP had a notable effect in the FAN available for fermentation (Fig. 3-C), the initial FAN concentration in BEP extrudate fermentation was 12.41-fold higher the concentration present in control experiment (1.8487 ± 0.0901 and 0.1485 ± 0.0052 mg FAN/ g SSB, respectively), thus showcasing the impact of BEP as significant pretreatment for enhancing bioavailability of the protein and amino acids available for yeast development and fermentation process. Control experiment presented a 13.13% increase in FAN concentration after 24 h of fermentation, this increment might be due to the scarce and challenging carbohydrate and protein concentrations in the SSB saccharified medium. These poor nutrient conditions and the consumption of cellobiose in BEP + ES experiment might be indicatives of a triggering of *P. kudriavzevii* cellulolytic pathway that might explain FAN increasing between the 24th and 48th hours of fermentation, previously the yeast cellulolytic capabilities in lignocellulosic matrices processing for ethanol production have been evaluated using wheat straw and cocoa husk (Jácome et al. 2024; Poomani et al. 2024). Previously a conjunction of BEP and bioconversion of buckwheat helped to enhance fungal cellulase activity, BEP extruded buckwheat presented a 1.5-fold increase in cellulase activity, therefore increasing the cellulose degradation rate. BEP mechanically affects lignin recalcitrant structure by making it more accessible for removal by *I. lacteus* fungus, thus preventing its inhibitory effect on fungal cellulase (Cao et al. 2023).

The type of pretreatment, fermentation time and their interaction had a significant effect on glucose concentration during the 72-h ethanolic fermentation (Table 6). After 72 h fermentation glucose was the only monosaccharide completely consumed by *P. kudriavzevii* in BEP + ES fermentation experiment, while in the control experiment, only 91.62% of glucose was consumed and 89.1% of it was consumed during the first 24 h of fermentation. Glucose concentration presented a significant inverse correlation with ethanol concentration and strong direct correlation with FAN concentration (Table 7). The difference in glucose consumption can be related to the difference in FAN concentration in the fermenting hydrolysate media, previous reports have shown that yeast biomass development and ethanol production is severely affected by the amino acid concentration present in the lignocellulosic liquor, previously increments of 1% v/v (from 0.5 to 1.5% L-proline) of the amino acid source on the growth media of *P. kudriavzevii* generated 65% of increment in the final ethanol concentration after a 24 h fermentation (Astuti et al. 2018). Yeast growth and fermentative metabolism is greatly affected by a nitrogen source limiting conditions, this can lead to residual glucose on media, lower substrate inhibition resistance and higher fermentation time requirements resulting in lower productivities and fermentation inhibitor resistance, particularly in the case of *Saccharomyces cerevisiae* and *P. kudriavzevii* (Díaz-Nava et al. 2017). In this study an increase of 92% in FAN concentration resulted in a 88% increase in ethanol productivity (from 0.008 g L^-1^ h^-1^ to 0.072 g L^-1^h^-1^), a higher increase when compared to previous reports obtaining a 72% using *P. kudriavzevii* growing enriched basal media.

**Table 6:** Analysis of variance of the selected independent variables for the evaluated responses in ethanolic fermentation using *P. kudriavzevii*

Factor	Glu		Xyl		MA		Cellobiose		FAN		EtOH	
	p-value	R^2^	p-value	R^2^	p-value	R^2^	p-value	R^2^	p-value	R^2^	p-value	R^2^
Type of pretreatment	< 0.001**	0.9955	< 0.001**	0.9912	< 0.001**	0.9850	< 0.001**	0.9935	< 0.001**	0.9947	< 0.001**	0.9689
Fermentation time (h)	< 0.001**		0.019*		0.190		0.124		< 0.001**		< 0.001**	
Interaction	< 0.001**		0.012*		0.184		0.124		< 0.001**		< 0.001**	

* Significant at *p* < 0.05; ** highly significant at *p* < 0.01

**Table 7:** Correlation matrix for the response variables from SSB ethanolic fermentation experiments

	Glu	Xyl	MA	Cel	EtOH
Xyl	0.451				
MA	0.463	0.971*			
Cel	0.410	0.994*	0.982*		
EtOH	-0.571*	0.431	0.376	0.452	
FAN	0.553*	0.974*	0.982*	0.979*	0.286

Correlation significant at *p* ≤ 0.05

Residual cellulose derivatives, as cellobiose, were detected only in the BEP + ES fermentation experiment; no cellobiose was detected in control experiment. In BEP + ES fermentation the cellobiose concentration decreased 14.37% after 48 h of fermentation, to later increase a 13.54% to reach a concentration with no significant difference when compared to the initial one (*p* > 0.05). Cellobiose concentration presented a significant direct correlation with FAN, Xylose and MA concentration (Table 7).

Hemicellulose components such as Xylose and Mannose + Arabinose (MA) concentration were significantly impacted by the type of pretreatment and fermentation time during the 72-h ethanolic fermentation (Table 6). Xylose content decreased during the 72-h fermentation, in BEP extrudate fermentation only 14.4% of the initial xylose was consumed during the first 48 h, to later increase its concentration at 72 h to 53.73 ± 0.18 mg Xyl/ g SSBs, in contrast 38.12% of xylose was consumed in control fermentation after the 72-h fermentation. Xylose consumption presented a significant direct correlation with FAN concentration (Table 7). Previously (Poomani et al. 2023) reported a 3.17 mg/mL ethanol production fermenting at 41 °C using alkaline pretreated wheat straw with a xylanase producing wild *P. kudriavzevii* strain isolated from cow rumen, they state that the pretreatment of the lignocellulosic matrix is important to enhance the xylanase and cellulase sugar release efficiencies and in addition the ethanol production by the yeast, in this study the importance of the pretreatment used on the lignocellulosic matrix is also reflected in *P. kudriavzevii* production of 5.18 mg/mL in the BEP hydrolysate and 0.70 mg/mL in the control fermentation without BEP pretreatment; as stated in the pretreatment section, this might be related to the lignin redistribution on the SSB by the BEP, making cellulose and hemicellulose not only more susceptible to the enzymatic saccharification of the pretreatment enzymes but also to the *P. kudtriavzevii* xylanase and cellulase digestion.

Yeasts consume xylose usually under aerobic or semi-aerobic conditions, during the initial 24 h the flasks may still contain air while yeast cells produce CO_2_ to fully displace remnant air (Hoppert et al. 2022). No xylitol production was detected on these experiments, and as Fig. 3-A shows, during the first 48 h in BEP + ES pretreatment fermentation *P. kudriavzevii* ATCC 20,381 was capable of utilizing some of the xylose present in the media which indicates that the yeast might be assimilating xylose for biomass formation, whereas in ES fermentation (Fig. 3-B) in a more challenging media with limiting carbon and nitrogen sources, yeast cells keep consuming xylose up to the 72 h of fermentation probably for biomass formation as well, this due to the low ethanol production. Rare carbohydrate consumption might be related to carbon source preference and carbon source co-utilization phenomena which is an evolutionary trait and a strain specific phenomena, thus explaining this experiment results contrasting to previous reports where xylose consumption and ethanol production occurred when using pure xylose as carbon source(Nweze et al. 2021) and where no consumption or ethanol production was achieved by *P. kudriavzevii* ITV-S42 (isolated from sweet sorghum juice) using a semi-synthetic media with different carbon sources(Díaz-Nava et al. 2017)d *kudriavzevii* HOP-1 (isolated from sugarcane juice) using alkali-treated rice straw as a carbon source (Oberoi et al. 2012), thus more research is needed to asses xylose contribution to *P. kudriavzevii* ATCC 20,381 ethanol producing capabilities.

MA content in BEP + ES fermentation had a parabolic behavior, it decreased during the first 24 h fermentation 3.95% of the initial MA, to later increase 5.52% its concentration at 48 h, to finally at 72 h reach a 15.4% consumption of the MA media content. In the ES control experiment MA concentration increased 13.89% after 24 h fermentation to later decrease to initial concentration at 48 h and remained unchaged afterwards. MA consumption presented a significant direct correlation with FAN, xylose and cellobiose concentration (Table 5). Mannose and arabinose consumption correlation with xylose might be explained with P. *kudriavzevii* ability to degrade hemicellulose. As stated before, previous experiments have demonstrated the ability of several wild type isolated *P. kudriavzevii* strains to metabolize carbohydrates derived from hemicellulose from various plant matrices such as mannose (isolated from idli batter (Chelliah et al. 2016), xylose (isolated from Gentian mash (Hoppert et al. 2022) and arabinose (isolated from river water(Kodama et al. 2013). This carbohydrate consumption by *P. kudriavzevii* ATCC 20381 presents it as an excellent candidate for industrial ethanol production, this because its robust ability to assimilate several and different carbon sources during fermentation with practically no by-product, and its capability of metabolizing pentoses (xylose and arabinose), which can increase the overall ethanol production from lignocellulose by up to 25–30% based on the original carbohydrate (Ndubuisi et al. 2023). As with xylose, further research is needed to assess mannose and arabinose contribution to *P. kudriavzevii* ATCC 20381 ethanol producing capabilities.

After 72 h of fermentation, the carbohydrates in both experiments were consumed and transformed into 51.84 ± 1.82 and 5.99 ± 1.40 mg EtOH/ g SSB in BEP + ES and ES hydrolysates respectively. This ethanol production is a 17.29% increment when compared to traditional twin extrusion pretreatment fermentation of SSB extrudate. The ethanol conversion efficiencies were 36.65% and 19.12%, respectively. In the control experiment there were no significant differences in ethanol concentrations when 48 and 72 h fermentation times were compared, thus after 48 h fermentation, *P. kudriavzevii* used the remaining glucose and FAN to survive and didn’t synthesize a significant amount of ethanol. The nitrogen source, its concentration and amino acid composition can impact significantly the ethanol, biomass and carbohydrate consumption during yeast growth, including *Pichia kudriavzevii* strains(Roca-mesa et al. 2020; Schnierda et al. 2014). The resulting residual carbohydrate content left in ES fermentation can be related to the aforementioned low nitrogen concentration (Fig. 2). When comparing the BEP + ES media (richer in carbon sources and FAN) with the ES, it is clear that the control experiment does not meet the required nutrients concentration for a higher cell growth, ethanol production and consumption of the different carbohydrates present in the media. In this study the ethanol production was reduced 88% when using ES media, previous studies have shown that a compromised nitrogen source in composition, quality and concentration can reduce up to 50% the ethanol production when using a *P.kudriavzevii* strain (BB2) (Fernandes et al. 2020).Simultaneous saccharification fermentation could improve the time of process and efficiency even in monoculture or coculture with a complementary strain.

## Conclusions

This study highlights the importance of SC and T_LBZ_ in enhancing lignocellulosic biomass processing for bioethanol production. The BEP + ES effectively disrupted SSB structure significantly enhancing saccharification yields, leading to a threefold increase in sugar release when compared to ES control. Fermentation experiments with *Pichia kudriavzevii ATCC 20,381* demonstrated its ability to utilize glucose, xylose, MA, and cellobiose, making it a robust microorganism for ethanol production. BEP treated SSB exhibited higher free amino nitrogen (FAN) release, significantly affecting fermentation parameters, including ethanol yield, carbohydrate consumption, and microbial growth. The SSB pretreated with BEP + ES showed a 12.41-fold increase in FAN compared to control, and thus producing 8.81-fold the ethanol that the ES control synthetized. These results highlight the critical role of protein and amino acid release in enhancing microbial fermentation efficiency. These findings reinforce BEP as a powerful pretreatment strategy for enhancing lignocellulosic ethanol production through improved substrate accessibility.

## Data Availability

The datasets used and analyzed during the current study are available from the corresponding author on reasonable request.
